# Poly(A)-binding proteins are required for microRNA-mediated silencing and to promote target deadenylation in *C. elegans*

**DOI:** 10.1093/nar/gkw276

**Published:** 2016-04-19

**Authors:** Mathieu N. Flamand, Edlyn Wu, Ajay Vashisht, Guillaume Jannot, Brett D. Keiper, Martin J. Simard, James Wohlschlegel, Thomas F. Duchaine

**Affiliations:** 1 Department of Biochemistry, McGill University, Montreal, QC H3A 1A3, Canada; 2 Goodman Cancer Research Center, McGill University, Montreal, QC H3A 1A3, Canada; 3 Division of Experimental Medicine & Goodman Cancer Research Center, McGill University, Montreal, QC H3A 1A3, Canada; 4 Department of Biological Chemistry David Geffen School of Medicine at UCLA, Los Angeles, CA 90095, USA; 5 St-Patrick Research Group in Basic Oncology, Centre Hospitalier Universitaire de Québec-Université Laval (Hôtel-Dieu de Québec), Laval University Cancer Research Centre, Quebec City, QC G1R 2J6, Canada; 6 Department of Biochemistry and Molecular Biology, Brody School of Medicine at East Carolina University, Greenville, NC 27834, USA

## Abstract

Cytoplasmic poly(A)-binding proteins (PABPs) link mRNA 3′ termini to translation initiation factors, but they also play key roles in mRNA regulation and decay. Reports from mice, zebrafish and *Drosophila* further involved PABPs in microRNA (miRNA)-mediated silencing, but through seemingly distinct mechanisms. Here, we implicate the two *Caenorhabditis elegans* PABPs (PAB-1 and PAB-2) in miRNA-mediated silencing, and elucidate their mechanisms of action using concerted genetics, protein interaction analyses, and cell-free assays. We find that *C. elegans* PABPs are required for miRNA-mediated silencing in embryonic and larval developmental stages, where they act through a multi-faceted mechanism. Depletion of PAB-1 and PAB-2 results in loss of both poly(A)-dependent and -independent translational silencing. PABPs accelerate miRNA-mediated deadenylation, but this contribution can be modulated by 3′UTR sequences. While greater distances with the poly(A) tail exacerbate dependency on PABP for deadenylation, more potent miRNA-binding sites partially suppress this effect. Our results refine the roles of PABPs in miRNA-mediated silencing and support a model wherein they enable miRNA-binding sites by looping the 3′UTR poly(A) tail to the bound miRISC and deadenylase.

## INTRODUCTION

MicroRNAs (miRNAs) are 18- to 25-nucleotide-long RNAs that regulate gene expression and impact on a wide variety of biological functions ranging from development to disease (1). In metazoans, miRNAs imperfectly base-pair with the 3′-untranslated regions (3′UTRs) of mRNAs. Target recognition instigates an intertwined series of silencing mechanisms, which includes mRNA translational repression, deadenylation and decay (2–9). The gene-silencing functions of miRNAs are orchestrated through interactions and activities of the miRNA-induced silencing complex (miRISC) and its associated co-factors. miRISC functional architecture revolves around the core Argonaute and GW182 proteins. In mammals and flies, the GW182 proteins encode an Argonaute-binding domain characterized by GW/WG repeats (10), and a silencing domain (SD) which interacts with CNOT1, a scaffold protein of the multi-subunit CCR4/NOT1 deadenylase complex (8,11–13). As such, the GW182 proteins bridge miRNAs to effector silencing machineries. The *Caenorhabditis elegans* homologs of GW182, the AIN-1 and AIN-2 proteins, strikingly diverge from vertebrate and insect homologs at the primary sequence level, harbor fewer copies of the GW motif, and do not encode distinctly recognizable domains. Yet AIN-1 and AIN-2 interact with the Argonaute proteins ALG-1 and ALG-2 and are thought to be essential for miRNA-mediated silencing (14). Surprisingly, recent data indicate that cross-species interactions are possible between Argonautes, GW182 proteins and CCR4/NOT1, when reconstituted in heterologous systems (15,16).

A growing number of accessory proteins have been identified through genetics (17–19) and molecular interactions (14) that are required for or promote miRNA-mediated silencing. Because co-factors were characterized using distinct experimental strategies and models, the universality of their roles in miRNA-mediated silencing across contexts and species is often unclear. This is the case for the function of cytoplasmic poly(A)-binding proteins (PABPs). Whereas experimental insight from *in vitro* translation extracts from mouse ascites cells indicate that it is essential for deadenylation (5), it appeared to only promote deadenylation without being absolutely required in flies (20). Another report proposed that PABP could help recruit miRISC to targeted mRNAs (21), whereas others suggested that PABP is displaced from mRNAs by miRISC (22). Clearly, at present, the roles for PABP in general translation initiation, poly(A) tail binding and protection, and its co-factor function with deadenylases are still conceptually entangled with the multi-layered mechanism of miRNA-mediated silencing.

Here, we examined the functions of the two *C. elegans* PABP orthologs in miRNA-dependent silencing using an integrated approach of genetics and biochemistry. We show that PAB-1 and PAB-2 physically and genetically interact with miRNAs and the miRISC machinery in embryonic and larval developmental stages. Biochemical depletion of PAB-1 and PAB-2 impinged on deadenylation of miRNA reporters but this function was modulated by the constitution of 3′UTRs. Finally, depletion resulted in loss of all translational silencing, independently of the presence of the poly(A) tail. We further resolve the critical roles of PABPs in miRNA-mediated silencing, and provide substantiated interpretations to the apparent discrepancies on its function that prevail in the literature.

## MATERIALS AND METHODS

Worm strains used: N2 Bristol (WT), *pab-2* (ok1851), *let-7* (n2853), MH2636 (*otIs114*(Plim-6::GFP, rol-6(d)), *lsy-6*(ot150)), FD01(*pab-2*(ok1851), *otIs114*(Plim-6::GFP, rol-6(d)), *lsy-6*(ot150)), FD02(*pab-2*(ok1851), *otIs114*(Plim-6::GFP, rol-6(d))). All strains were grown at 22°C except *let-7*(n2853), which was maintained at 16°C.

### Preparation of embryonic extracts

Embryonic extracts were prepared as in (23) with the only difference that calf liver tRNA was omitted preparations without any sensible difference in outcome.

***In vitro* transcription, mRNA stability, translation and deadenylation assays** performed as described in (9). Half-deadenylation times were calculated by determining the intersect of the non-deadenylated and deadenylated RNA species over time using polynomial regression (order 2) (R Project or Microsoft Excel), using quantification of autoradiography with ImageJ. PAB-1/2 were depleted using GST-PAIP2 as in (24).

### 2′-*O*-Methyl (2′-*O*-Me) pull-down

N2 embryos were homogenized in two volumes of lysis buffer (25 mM Hepes-KOH pH 7.4, 150 mM NaCl, 1 mM EDTA, 1 mM DTT, 10% glycerol, 0.5% Triton X-100 and protease inhibitors) using 30 strokes from a stainless steel homogenizer. S10 Worm lysate was pre-cleared with 25 μl of T1 streptavidin beads (Invitrogen) and non-specific 2′-*O*-Me oligonucleotides (miR-1, 10 pmol) for 1 h at 4°C with rotation. The supernatant was incubated with biotinylated 2′-*O*-Me oligonucleotides (10 pmol) and poly(A)_25_ RNA (10 pmol) for 1 h at 22°C. The extract was spun at maximum speed for 5 min in a tabletop centrifuge. The supernatant was then incubated with 50 μl of T1 streptadividin beads for 30 min at 4°C. Beads were washed three times using ice-cold lysis buffer containing 0.1% Triton X-100 and 2 mM DTT, followed by a wash without detergent and 2 mM DTT. Beads were resuspended in 50 μl of 2× sodium dodecyl sulphate (SDS) loading buffer and eluted by heating at 95°C for 4 min. One fifth of the proteins was loaded on gel and analyzed by western blot. For mass spectroscopy, the pull-down was scaled up 5-fold, and two additional washes without Triton X-100 were performed. Beads were frozen on dry ice and analyzed by MuDPIT. Multidimensional Protein Identification (MuDPIT) was performed as described in (25).

### GST-pulldown

Embryos were homogenized in three volumes of lysis buffer (25mM Tris–Cl pH 7.5, 100 mM KCl, 2.5 mM MgCl_2_, 0.1% Triton X-100, 10% glycerol, 0.1ng/μl RNaseA) using 30 strokes from a stainless steel homogenizer. A total of 2 mg of S10 lysate was incubated with 50 μl of Gluthatione-Sepharose 4B beads (GE healthcare Life Sciences) pre-coupled to 100 µg of GST-PAIP2 or GST recombinant protein for 1 h at 4°C. Beads were washed 4 times in lysis buffer for 5 min at 4°C and bound proteins were eluted in 2× SDS loading buffer and analyzed by sodium dodecyl sulphate-polyacrylamide gel electrophoresis (SDS-PAGE) and western blot.

### RNA interference

RNAi was performed as in (26) for injections and as in (27) for feeding. The genomic sequence of *pab-1* was amplified using the following primers: (1896) TAATACGACTCACTATAGGGCATTACCAGCTGCGAAGTCA and (1897) TAATACGACTCACTATAGGGTGTGTCCCGGGTAGAGTTTC, while the following primers were used for *pab-2*: (1894) TAATACGACTCACTATAGGGGGAAGGAACTGACTGCAAGC; (1895) TAATACGACTCACTATAGGGCCTCGACCTTGCTTCTGAAC. Polymerase chain reaction (PCR) products were cloned in pSC-A-amp/kan and transformed in HT115. L1 animals were plated and placed at 16°C. The next generation of animals (F1) was assayed for bursting. For RNAi by injection, the RNA was synthesized using the T7 MegaScript kit (Ambion) using PCR products as templates. The RNAs were purified with mini quick spin RNA columns (Roche), precipitated with ethanol and resuspended in H_2_O. L4 worms were injected in the head or tail body cavity with 100 ng/μl of dsRNA. The next generation of animals (F1) was assayed for bursting.

### Western blotting

**Figure 1.**
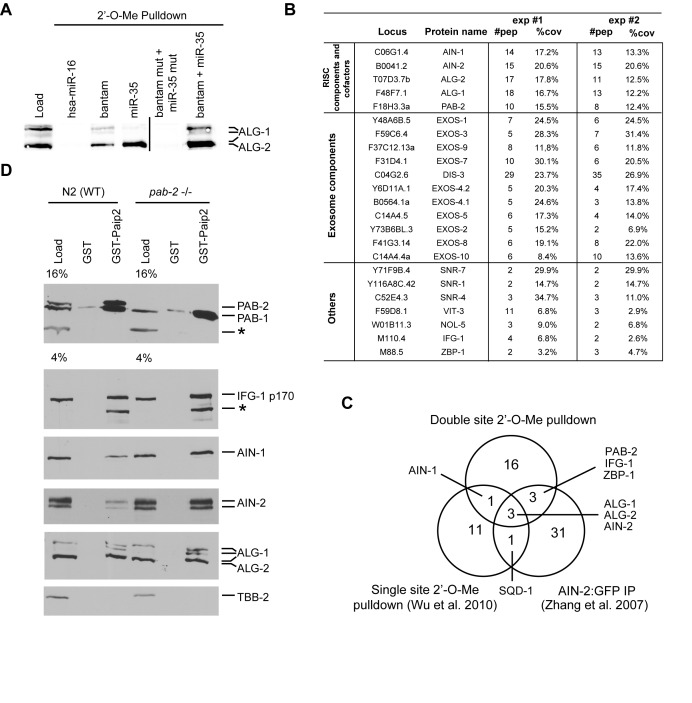
PAB-1 and PAB-2 interact with miRISC in *Caenorhabditis elegans* embryos. (**A**) ALG-1/2 western blot analysis of 2′-*O*-Me pull-downs. 5′ Biotinylated 2′-*O*-Me oligos containing miR-35, CeBantam or both sites were used to pull down the miRISC WT strain (N2) embryonic extract. ALG-1 and ALG-2 were detected using near-infrared fluorescent western blot (LiCOR). (**B**) Proteins identified in MuDPIT analyses of a dual site (CeBantam + miR-35) 2′-*O*-Me pull-down. Only proteins which were detected in two independent purifications and were absent from pull-downs with mutated binding sites are included here. For each protein, the number of peptides (#pep) found in each experiment and the coverage (%cov) of these peptides for the full-length protein are indicated. (**C**) Venn diagram comparing detected interactions with the dual site (CeBantam + miR-35) pull-down to single-site RISC pull-down, and AIN-2-GFP IP. (**D**) A GST pull-down using either GST or GST-PAIP2 was performed either on WT (N2) or mutant *pab-2(0) (ok1851)* embryonic extract in the presence of 0.1 ng/μl RNAse A. The bound proteins were analyzed on SDS-PAGE and detected by western blot.*: non-specific band.

The PAB-1/2 polyclonal antiserum was raised against the C-terminal region of PAB-2 by injecting rabbits with the following peptide: EIDNAELIMMLQDAELFRSKVEEAFGV. Serum is used at a 1:3000 dilution in 5% non-fat dry milk in Tween 0.1%-PBS O/N at 4°C. For quantification of depletion, 5 μl (PAB, ALG-1/2) or 2.5 μl (TBB-2, AIN-1, AIN-2) of treated extract was mix with 2× SDS loading buffer and loaded on an 8% SDS-PAGE gel. Signal for chemiluminescent western blot was acquired with film (Figure 1D) or using the FluorChem HD2 system (Figure 3). Signal for fluorescent western blot (Figure 1A) was acquired with the LI-COR Odyssey imaging system.

### Plasmid constructs

The constructs harboring 3 or 6× miR-35 sites or miR-52 sites and a 161 nt linker were previously generated in (9). To generate the pCI-RL-6×-miR-35-pA constructs with a short linker (L32), the pCI-RL-6×-miR35-pA plasmid was digested with XbaI/NotI (NEB) and the insert was cloned in the pCI-RL-L32-pA backbone. To generate the construct with the long linker (L262), the linker pCI-RL was amplified using the primers: Tdo761 (GCGGCCGCTTCCCTTTAG) and Tdo762 (GCGGCCGCAATAAAGCATTTTTTTCACTGCA) and cloned in the NotI site of pCI-RL-6×-miR-35-pA.

## RESULTS

### PAB-1 and PAB-2 associate with miRISC in *C. elegans* embryos

We previously conducted a comparative proteomic analysis of miRISC captures using pull-down of 2′-*O*-methylated (2′-*O*-Me) oligonucleotide mimics of single target sites for maternal and zygotic miRNA families highly enriched in the embryo (9,28,29). As single miRNA-binding sites do not trigger target deadenylation (9), we reasoned that capture based on mimics of potent combinations of target sites would better reflect components of active miRISC. We thus performed miRISC capture using a mimic of a pair of miRNA-binding sites for the bantam and miR-35-42 miRNAs, in a configuration that is highly active in deadenylation assays (Flamand *et al*., in preparation). Western blot analysis indicated that both single and dual sites baits efficiently pulled down the miRNA-dedicated Argonaute proteins ALG-1 and ALG-2 (Figure 1A), whereas control baits, such as a dual-site oligonucleotide with mutated seed-binding sequences or for the non-related human miR-16 (hsa-miR-16), did not. Two independent large-scale affinity purifications were conducted, and the recovered fractions were subjected to multi-dimensional protein identification technology (MuDPIT) analysis (30). Only interactions consistently identified in independent captures were further considered, and proteins captured with the mutated bait were excluded from the interaction list (Figure 1B). As expected, known core miRISC components ALG-1, ALG-2, AIN-1 and AIN-2 were detected. In addition, PAB-2, eleven subunits of the exosome complex, eIF4G homolog IFG-1, hnRNP K family protein ZBP-1, NOL-5 and proteins often detected in unrelated *C. elegans* MuDPIT analyses (three SNR proteins and VIT-3) were also detected.

Core miRISC components ALG-1, ALG-2 and AIN-2 had also been detected in single target site captures (9) and in a proteomic survey of AIN-2-GFP immunoprecipitates (IP) (14), whereas AIN-1 was detectable in single- and double-miRNA-binding site captures, but not in AIN-2-GFP IP (Figure 1C). PAB-2, the exosome complex components, IFG-1 and ZBP-1 had not been detected in our previous single target mimic captures (9).

To confirm the interaction of PAB-2 with the miRISC, we turned to a distinct strategy of co-purification. To specifically pull down PAB-1 and PAB-2, we exploited the mammalian PABP interacting protein 2 (PAIP2) (31). As PAIP2 was shown to deplete PABPs from murine extract (24) and fly embryonic extract (20), we reasoned that it could also interact with *C. elegans* PAB-1 and PAB-2. PAB-1 and PAB-2 share 66% identity, 75% similarity with each other and 55–59% sequence identity, 67% similarity with hsPABPC1 (Supplementary Figure S1). Indeed, western blotting using an antibody specific to PAB-1 and PAB-2 (described in Supplementary Figure S2A) indicated that the GST-PAIP2 fusion could efficiently pull down PAB-1 and PAB-2 from embryonic lysates, independently of RNA (Figure 1D). Core miRISC components ALG-1, ALG-2, AIN-1 and AIN-2 were specifically detected in the pull-down fractions by western blot. Furthermore, GST-PAIP2 also pulled down IFG-1, an expected interactor of PAB-1 and PAB-2, thus confirming our MuDPIT analyses. To test if this interaction also occurs with the PAB-1 paralog, we performed pull-down from *pab-2(ok1851)* embryonic extract, in which PAB-2 is genetically depleted. PAB-1 co-purified with miRISC components as efficiently as when both PAB-1 and PAB-2 were present (Figure 1D, *pab-2* −/− lanes). Treatment with RNase A or Micrococcal nuclease (MNase) did not impinge on co-purification with miRISC, confirming an RNA-independent interaction (Supplementary Figure S2). Together with proteomics analyses, these results indicate that, as in mammals and flies, *C. elegans* embryonic miRISC interacts with the PABPs PAB-1 and PAB-2 *in vivo*.

### *pab-1* and *pab-2* genetically cooperate with miRNAs

**Figure 2.**
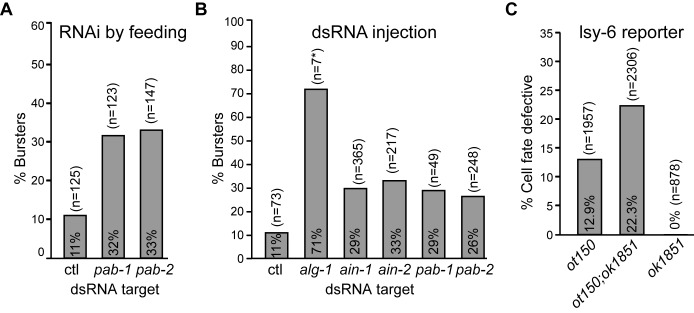
*pab-1 and pab-2* genetically cooperate with *let-7* and *lsy-6* miRNAs. (**A**) *pab-1* and *pab-2* genetically interact with the *let-7* miRNA. *let-7(n2853)* animals (L1) were fed with bacterially-expressed dsRNA against the indicated gene, or L4 animals injected with dsRNA (**B**) and F1 animals were scored for bursting vulva phenotype at the permissive temperature (16°C). Results shown are representative of at least two experimental and biological replicates. (**C**) *pab-2* genetically interacts with *lsy-6. pab-2(0)(ok1851)* were crossed with OH3646 (*otIs114(Plim-6::GFP, rol-6(d)*);*lsy-6(ot150)*. Animals were scored for expression of GFP in the ASEL.

*let-7* was previously implicated in the translation repression of its targets during the larval developmental stages of *C. elegans* (32). To examine the role of PAB-1 and PAB-2 and other proteins identified in our proteomic survey in miRNA-mediated translation repression, we tested their genetic interaction with *let-7*. As previously noted, complete genetic depletion of *pab-1* and *pab-2* leads to sterility, due to germline proliferation defects and pleiotropic effects (33,34); we thus employed a sensitized genetic assay based on the temperature-sensitive *let-7(n2853)* hypomorphic allele. This mutant exhibits a temperature-sensitive L4-to-adult transition defect, which results in a bursting vulva phenotype (35). This visible outcome nears full penetrance at non-permissive temperature (20 and 25°C), and appears at an approximate incidence of 10–15% at permissive temperature (16°C) (Figure 2A and B). Bursting was recently attributed to the mis-regulation of a single target, *lin-41*, as mutating a single *let-7* binding site in *lin-41* 3′UTR recapitulates the phenotype (36). To induce a strong knock-down, L1 larvae animals were exposed to RNAi by feeding dsRNA-expressing bacteria (27) (Figure 2A) or L4 larvae were injected with candidate-specific dsRNA (26) (Figure 2B), and the progeny (F1) was scored for the bursting vulva phenotype at the permissive temperature (16°C). Among candidates identified in the MuDPIT analysis, only core miRISC components and *pab-1* and *pab-2* exacerbated the bursting phenotype (Figure 2A, B and Supplementary Figure S3). Depletion of either *pab-1* or *pab-2* by feeding or dsRNA injection enhanced the bursting phenotype to the same extent as *ain-1* or *ain-2* RNAi. RNAi against *pab-1* or *pab-*2 using the feeding method resulted in 32 and 33% bursting animals, respectively, compared to 11% in control RNAi (Figure 2A). By injection, bursting was increased to 29 and 26% respectively for *pab-1* and *pab-2*, and to 29 and 33% for miRISC components *ain-1* and *ain-2*, respectively (Figure 2B, Supplementary Figure S3). In comparison, knockdown of the miRNA-dedicated Argonaute *alg-1* resulted in larval arrest and strong incidence of bursting (∼70%). These results demonstrate that *pab-1* and *pab-2* are required for the full function of *let-7* in larval development.

To determine the importance of *pab-2* in embryonic miRNA-mediated silencing, we tested the genetic interaction of the *pab-2(ok1851)* null allele with a miRNA involved in an early developmental decision. As *pab-1* null alleles are sterile (33,34), the same assays could not be performed with this paralog. During embryogenesis, the *lsy-6* miRNA controls ASE cell fate differentiation into left (ASEL) and right (ASER) asymmetric neurons (37). In ASEL, *lsy-6* suppresses *cog-1*, itself a suppressor of ASEL cell fate (37). The p*lim-6*::GFP reporter specifically drives expression of GFP in ASEL, enabling quantification of *lsy-6* function and associated miRISC components by visualizing ASE cell fate (17,38,39)(Supplementary Figure S3). Whereas a null allele of *lsy-6* results in 100% ASEL to ASER transition, the *lsy-6(ot150)* hypomorphic allele displays ∼15% cell fate change. *pab-2(ok1851)* led to significant loss of ASEL in this strain. Transition was observed in 22.3% of *pab-2(ok1851)*; *lsy-6(ot150)* cells compared to 12.9% in *lsy-6(ot150)* alone (Figure 2C). This phenotype was determined through interaction with *lsy-6*, as *pab-2*(*ok1851)* alone did not display any detectable cell fate transition. These results show that *pab-2* is important for the function of *lsy-6* in determining ASEL cell fate during embryogenesis. Together, results from the *let-7* and *lsy-6* genetic assays confirm that *pab-1* and *pab-2* are important for effective silencing by miRNAs *in vivo*.

### PAB-1 and PAB-2 promote, but are not absolutely required for miRNA-mediated deadenylation

**Figure 3.**
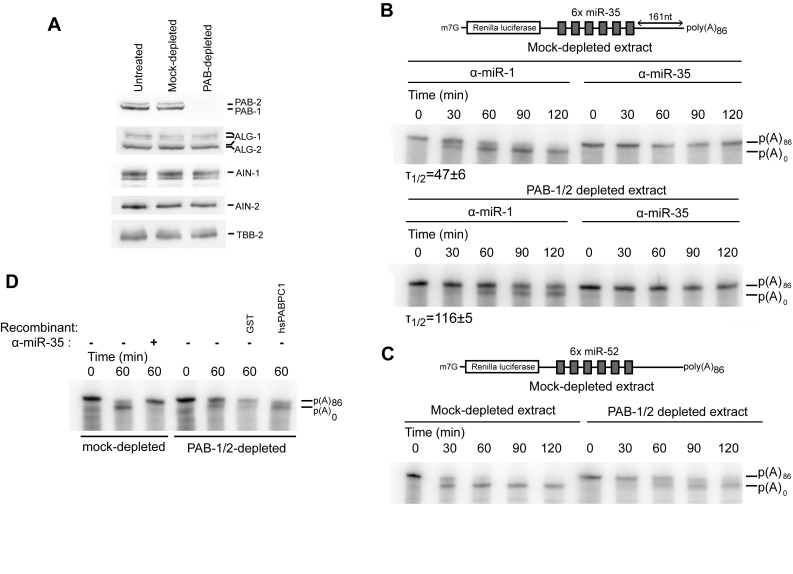
Biochemical depletion of PAB1 and PAB-2 delays deadenylation. (**A**) PAB-1 and PAB-2 levels were assessed by western blot in GST- or GST-PAIP2-treated extracts. A fraction of the extract was analyzed by SDS-PAGE followed by western blotting. (**B**) RL-6×-miR-35-p(A)_86_ was subjected to an *in vitro* deadenylation assay in the presence of a miR-35 or miR-1 2′-*O*-Me inhibitor in GST- or GST-PAIP2-treated extracts. RNA was extracted and analyzed by UREA-PAGE. (**C**) RL-6×-miR-52-p(A)_86_ was subjected to an *in vitro* deadenylation assay in GST- or GST-PAIP2-treated extracts. RNA was extracted and analyzed by UREA-PAGE. (**D**) Human PABC1 restores deadenylation in the *Caenorhabditis elegans* embryonic extract. PAB-1/2-depleted extract was supplemented with 115 nM of human PABC1 or GST proteins and a radiolabeled RL-6×-miR-35-p(A)_86_ was subjected to deadenylation and analyzed by UREA-PAGE. *T*_1/2_ is the half deadenylation time (min).

To precisely investigate how PAB-1 and PAB-2 function in miRNA-mediated silencing in *C. elegans*, we used an *in vitro* embryonic translation extract previously developed in our lab (9,23). An extract prepared from *pab-2(ok1851)* animals exhibited an overall reduced translation activity. Surprisingly, *pab-2* deletion left deadenylation of miR-35 reporter mRNAs unaffected (Supplementary Figure S4). Since PAB-1 levels remain unchanged in these animals (Supplementary Figure S2), we reasoned it could compensate for loss of PAB-2. Unfortunately, the most potent *pab-1(RNAi)* attempts in the *pab-2* strain could not deplete beyond a ∼2-fold reduction (Supplementary Figure S2) and led to sterility and small brood size, preventing the generation of a useful embryonic translation extract. To assess the mechanistic implications of both PAB-1 and PAB-2 depletion, we turned to a biochemical strategy using matrix-bound GST-PAIP2 on the embryonic extract. Using this strategy, the levels of PAB-1 and PAB-2 could be reduced below detection levels in the embryonic extract (Figure 3A, PAB-1/2-depleted) without depleting miRISC proteins ALG-1, AIN-1 and AIN-2, whereas a GST (mock-treated) matrix left the PAB-1 and PAB-2 levels unchanged. In PAB-1/2-depleted extracts, deadenylation of Renilla luciferase (RL) transcripts encoding six miR-35-binding sites and a poly(A) tail (RL-6×-miR-35-pA_86_) was slowed, but not blocked (Figure 3B; 6× miR-35). Whereas a mock-depleted extract reached half-deadenylation at 47 min, this point was reached at 116 min in PAB-1/2-depleted extract, representing a 2.5-fold decrease in deadenylation rate. Deadenylation of a similarly structured reporter encoding six binding sites for the zygotic miR-51-56 family was affected in a comparable manner by PAB-1/2 depletion (Figure 3C; 6× miR-52). When human PABC1 was added at 115 nM to the extract, it restored the deadenylation rate of the RL-6×-miR-35-pA_86_ reporter in the GST-PAIP2 treated extract (Figure 3D). Taken together, these results show that in *C. elegans* embryos, PAB-1 and PAB-2 promote miRNA-dependent deadenylation, but they are not absolutely required.

### PAB-1 and PAB-2 are essential for miRNA-mediated silencing

**Figure 4.**
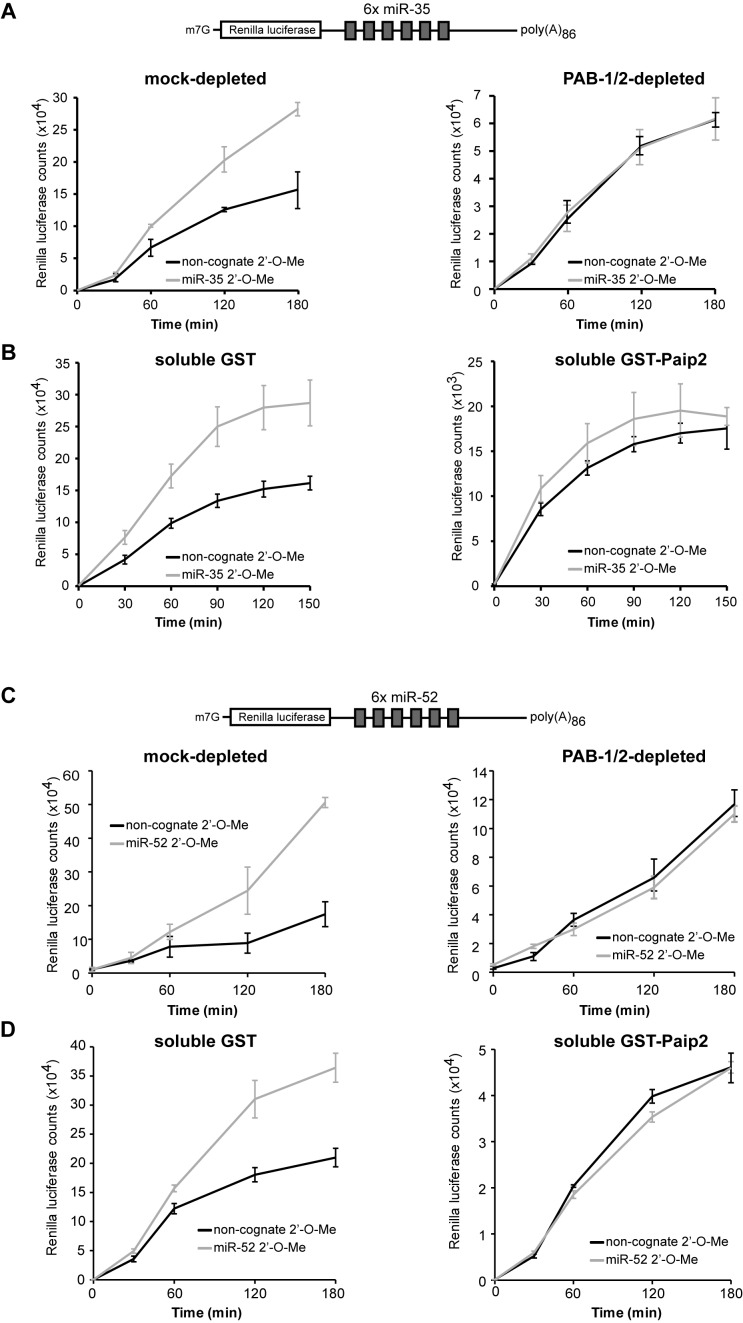
Depletion or impairment of PAB-1 and PAB-2 prevents miRNA-mediated silencing *in vitro*. The RL-6×-miR-35-p(A)_86_ reporter was subjected to a translational repression assay in GST- or GST-PAIP2-treated extracts (**A**) or in presence of 2.5μM soluble GST or GST-PAIP2 (**B**). Translational activity was monitored through measurement of RL activity in the presence of miR-35 2′-*O*-Me inhibitor or a non-cognate miR-1 2′-*O*-Me inhibitor. The RL-6×-miR-52-p(A)_86_ reporter was subjected to a translational repression assay in GST- or GST-PAIP2-treated extracts (**C**) or in presence of 5 μM soluble GST or GST-PAIP2 (**D**). Translational activity was monitored through measurement of RL activity in the presence of miR-52 2′-*O*-Me inhibitor or a non-cognate miR-58 2′-*O*-Me inhibitor.

We next investigated whether PAB-1 and PAB-2 play a role in miRNA-mediated silencing beyond accelerating target deadenylation. Overall, translation counts were diminished by ∼4-fold in PAB-1/2-depleted extracts compared to mock-depleted extracts (Figure 4A), and still yielded reliable RL activity. We found that miRNA-mediated translational silencing was entirely abolished in PAB-1/2-depleted extracts (Figure 4A). In untreated or mock-depleted extracts, a 2- to 4-fold increase in RL light counts was observed when a miR-35 2′-*O*-Me inhibitor was added prior to RL-6×-miR-35-pA_86_ reporter translation, in comparison with a non-cognate miR-1 2′-*O*-Me control (Figure 4A, left panel). Strikingly, no remaining miR-35-specific de-repression could be observed in PAB-1 and PAB-2-depleted extracts (right panel). Addition of soluble GST-PAIP2 to the extract, to block the activity of PABP as employed previously (20), led to the same outcome (Figure 4B). When the extract was pre-supplemented with 2.5 μM soluble GST-PAIP2, overall translation decreased by ∼9-fold, mimicking the consequences of PAB-1 and PAB-2 depletion. Furthermore, addition of 2.5 μM GST-PAIP-2 completely abolished miR-35-dependent translation silencing of the RL-6×-miR-35-pA_86_ reporter (right panel), whereas supplementation with 2.5 μM GST had no effect on miRNA-mediated repression (left panel). Again, assays performed using the reporter for the miR-51-56 family led to the same conclusions (Figure 4C and D). These results indicate that PAB-1 and PAB-2 are required for the entire process of miRNA-mediated translational silencing directed by *C. elegans* embryonic machinery.

**Figure 5.**
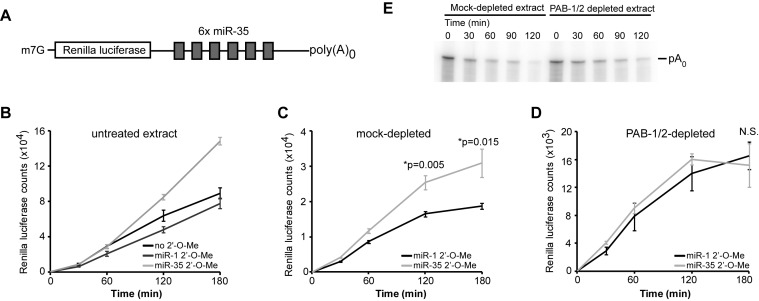
PAB-1 and PAB-2 are required for miRNA-mediated silencing independently of the poly(A) tail. (**A**) Design for the RNA used in the experiment. A capped transcript containing a Renilla luciferase ORF and six miR-35 binding sites in its 3′UTR, but lacking a poly(A) tail. (**B**) The reporter was incubated in an embryonic translation extract in the presence of 2′-*O*-Me inhibitor (miR-35) or non-specific inhibitor (miR-1). Translation activity was monitored through measurement of RL activity. Repression activity of the RL-6×-miR-35-p(A)_0_ transcript was assayed in (**C**) GST- or (**D**) GST-PAIP2-treated extracts. Translation counts were monitored over time in the presence of miR-35 or miR-1 2′-*O*-Me inhibitors. (**E**) RL-6×-miR-35-p(A)0 was incubated in GST- or GST-PAIP2-treated extracts. RNA was extracted and analyzed by UREA-PAGE. *: significance in a one-sided Welch *t*-test.

Our results demonstrate that PAB-1 and PAB-2 depletion, or their incapacitation, effectively un-couple the presence of a poly(A) tail from miRNA-mediated translation silencing. To further probe the mechanistic role of PAB-1 and PAB-2, we investigated whether the presence of a poly(A) tail is required for miRNA-mediated silencing and if PABPs require a poly(A) tail for their role in miRNA-mediated silencing. For this, we examined the translation of a RL-6×-miR-35 transcript lacking a poly(A) tail (RL-6×-miR-35-pA_0_) in the *in vitro* translation extract (Figure 5A). Consistent with PAB-1/2 depletion or inhibition, translation of the un-adenylated reporter was lowered by ∼4-fold in comparison with a reporter bearing a poly(A) tail. RL-6×-miR-35-p(A)_0_ reporter translation was specifically de-repressed when the extract was treated with a miR-35 2′-*O*-Me inhibitor, but not when supplemented with a non-cognate miR-1 control (Figure 5B). This result indicates that at least part of miRNA-mediated silencing in *C. elegans* embryo is independent of deadenylation or the presence of a poly(A) tail.

To address if PABPs are required for poly(A)-tail independent silencing, we performed reporter translation silencing assays on RL-6×-miR-35-pA_0_ in the PAB-1/2-depleted extract. In a mock-depleted extract, as in an untreated extract, the reporter was significantly de-repressed when treated with miR-35 2′-*O*-Me inhibitor, but not when treated with a non-cognate miR-1 inhibitor (Figure 5C). However, the same treatment did not de-repress the RL-6×-miR-35-pA_0_ reporter when translated in a PAB-1/2-depleted extract (Figure 5D). Repression of un-adenylated reporters was not due to *de novo* polyadenylation in the extract, as they did not undergo any increase in size (Figure 5E), and they could not be captured by oligo-dT-based qRT-PCR (Supplementary Figure S5). Together, these results show that the poly(A) tail is not required for all miRNA-mediated silencing in *C. elegans* embryo, and that PAB-1 and PAB-2 are essential for both poly(A) tail-dependent and -independent silencing aspects of the mechanism. These results further imply that PAB-1 and PAB-2 can act independently of their poly(A)-binding function in miRNA-mediated silencing.

### 3′ UTR primary structure modulate PABP contribution in miRNA-mediated deadenylation

**Figure 6.**
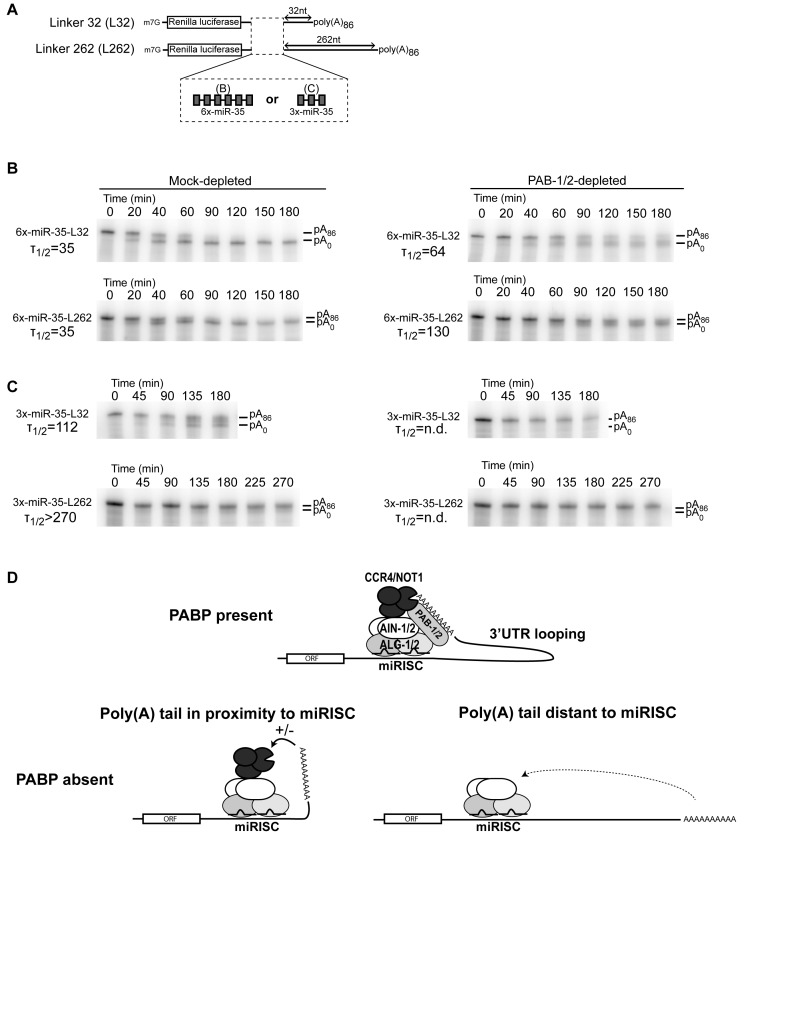
3′UTRs modulate PABP contribution in miRNA-mediated deadenylation. (**A**) Design of the RNAs used in the experiment. Capped transcripts encode the Renilla luciferase ORF and either three or six miR-35-binding sites in its 3′UTR, followed by a linker of 32 nt (L32) or 262 nt (L262). (**B**) RL-6×-miR-35-p(A)_86_ L32 and L262 were subjected to an *in vitro* deadenylation assay in GST- or GST-PAIP2-treated extract. (**C**) RL-3×-miR-35-p(A)_86_ L32 and L262 were subjected to an *in vitro* deadenylation assay in GST- or GST-PAIP2-treated extract. (**D**) The 3′UTR-poly(A) loop model of PABP function in miRNA-mediated deadenylation. PABPs enables miRNA-binding sites by facilitating access to the poly(A) tail to miRISC and to the associated deadenylase. Longer 3′UTRs exacerbate, and shorter 3′UTRs suppress PABP dependency. *T*_1/2_ correspond to the half deadenylation time (min).

We previously observed the impact of the 3′UTR landscape on miRNA target deadenylation rates (9). An increased distance between the miRNA-binding sites and the poly(A) tail negatively affected miRNA-directed deadenylation rates. We reasoned that PABPs may bridge the poly(A) tail to miRISC and its associated deadenylase (Figure 6). An important prediction based on this model is that miRNA-binding sites at greater distances from the poly(A) tail should be more sensitive to PABP depletion.

To test this, deadenylation assays were performed on RL-6×-miR-35-pA_86_ reporters bearing a shorter 32nt linker (L32), or a longer 262nt linker (L262) ahead of the poly(A) tail (Figure 6A). While both reporters were deadenylated at a similar rate in the control extract (Figure 6B, Mock-depleted; *T*_1/2_ 35 min), the two reporters were differentially affected by PAB-1/2 depletion. The L32 reporter was slowed by ∼2-fold (*T*_1/2_ 64 min), whereas the L262 reporter was slowed by ∼4-fold (*T*_1/2_ 130 min)(Figure 6B, PAB-1/2-depleted). Even though examined in different extract preparations, RL-6×-miR-35 reporters bearing an intermediate 161 nt linker exhibited an intermediate ∼2.5-fold delay under PAB-1/2 depletion (Figure 3B). These results suggest that PAB-1/2 contribution in miRNA-directed deadenylation is modulated by the distance between the target sites and the poly(A) tail.

Six copies of miR-35-binding sites exert a very potent drive toward reporter deadenylation in embryonic extracts. We next examined the impact of PABP depletion on a milder 3×-miRNA-binding site reporter. As expected, RL-3×-miR-35-pA_86_ reporters were deadenylated slower than 6× counterparts in the control extract (Figure 6B). However, RL-3×-miR-35-pA_86_ L262 was deadenylated much slower (>270 min) than its shorter linker version (L32; 112 min). Strikingly, the deadenylation of both L32 and L262 3× reporters were fully impaired in PAB-1/2 depleted extract. This result indicates that lesser miRNA-binding sites potency can exacerbate PAB-1/2 requirement in miRNA-mediated deadenylation.

Taken together, these results show that 3′UTR primary structure parameters, such as length and binding site potency, can modulate PAB-1/2 dependency in miRNA-mediated deadenylation. It furthermore substantiates a model wherein PABPs enables miRNA-mediated deadenylation by looping the poly(A) tail substrate to the miRISC-associated deadenylase (Figure 6D).

## DISCUSSION

In this study, multi-pronged genetic and biochemistry approaches indicate that PAB-1 and PAB-2, the *C. elegans* orthologs of human cytoplasmic PABPC1, interact physically and functionally with miRISC. PAB-1 and PAB-2 function in miRNA-mediated silencing was assessed not only using artificial miRNA reporters, but by examining the functional outcome of regulation on endogenous targets, embedded in two phenocritical genetic cascades. Probing mechanistic bases using embryonic cell-free assays uncovered a multi-faceted mechanism: depletion of PABPs beyond detectable levels prevented repression of polyadenylated and un-adenylated mRNA reporters, yet still permitted miRNA-mediated deadenylation. Furthermore, we show that 3′UTR-specific features, such as miRNA-binding site potency and their distance with the poly(A) tail, can modulate the contribution of PABPs in target deadenylation. Altogether, our findings substantiate a model wherein PABPs loop the poly(A) tail to target site-bound miRISC and its associated deadenylase, and offer avenues to reconcile the apparent discrepancies that stem from other reports.

With this study, the role of PABPs in miRNA-mediated silencing has now been examined in mice, *Drosophila*, zebrafish and *C. elegans*. Superficially, one may have expected that conserved machineries involved in biologically essential processes, such as basic translation and widespread gene regulation, should retain their purpose through phylogeny. It may be striking, then, that findings in the other species thus far have led to diverging conclusions on the role of PABPs in miRNA function. For example, mouse PABP is required, whereas in zebrafish PABP appears dispensable for at least some facets of miRNA-mediated silencing (40). Muddying the issue even further, in *Drosophila melanogaster* embryonic extracts, PABP promotes deadenylation but is not strictly required (20). An important challenge to resolving the role of PABPs in miRNA-mediated silencing across species thus lies in discerning between divergent interpretations that may be due to experimental designs, from the more biologically significant differences between species, and context-specific particularities. With this in mind, it is particularly enlightening that closely-related experimental designs based on PAIP2-depletion and incapacitation of PABPs have now been utilized in three out of four of those species, thus offering a valuable framework for comparison (5,20,21). Although leading to varying degrees of depletion, the different mechanistic outcomes established in these model systems cannot simply be reconciled by differences between experimental designs. Efficient depletion of PABPC1 from murine extract (>90%) (5) resulted in a complete block of deadenylation. In *Drosophila*, depletion using the same approach remained limited (∼20%) in the absence of MNase treatment. However, addition of soluble GST-PAIP2 resulted in a robust block of PABP activity, but nonetheless did not prevent deadenylation of a miRNA reporter (20,21). An interesting observation in the latter system is that translational silencing in the absence of poly(A) tail was disrupted neither by PABP depletion nor GST-PAIP2 addition. Another important piece of evidence comes from the role of PABP in zebrafish embryos. Experiments in this system did not rely on PAIP2-depletion of PABPs, but rather on potent morpholino oligonucleotide incapacitation of PABP expression. Using this strategy, Pabpc1 was depleted *in vivo* to <5%, yet repression of a miR-430 reporter was unaffected, leading to the suggestion that Pabpc1 is dispensable for silencing in zebrafish embryos (40).

Our results provide a straightforward explanation for such apparent discrepancies. The 3′UTRs examined in those reports have distinct structural features, have distinct qualities of binding sites and carry different number of copies. Furthermore, distinct expression levels and distinct stoichiometric ratio with experimental reporters likely also modulate their drive toward deadenylation. As such, and in light of our results, it is not surprising that they should exhibit distinct dependencies toward PABP. Accordingly, deadenylation of a 6×-let-7 reporter reached half-completion at ∼3 h in mouse ascites and was fully impaired by PABP depletion (5), whereas a 8x-let-7 reporter was deadenylated much faster in *Drosophila* lysates (*T*_1/2_ <30 min), and did not require PABP (20). We note that an important physiological corollary to this interpretation is that miRNA-binding sites located in different portions of 3′UTRs will employ PABP, while others may not require it. Furthermore, the functions of miRNAs in cellular contexts like neurons, wherein 3′UTRs can be extremely long (41), should be particularly sensitive to PABP availability. In a related manner, it was suggested that lowered PABP levels in different cellular contexts or in experimental settings can potentially modulate the effect of miRNAs by changing rate-limiting steps in translation (42). Finally, it is conceivable that yet another layer of 3′UTR-specific regulation will be contributed by folding of mRNA sequences into secondary and tertiary structures. While we have not specifically examined its impact on our reporters here, it will be interesting to examine how physiological 3′UTR structures enhance or impinge on miRNA-mediated deadenylation.

Another non-mutually exclusive possibility is that whereas the interaction of PABP with miRISC, as well as its involvement in miRNA-mediated silencing have been retained, their molecular underpinnings have been considerably and continuously reorganized throughout evolution. This possibility is suggested by the striking structural plasticity of GW182 miRISC scaffold proteins across species. Human and mouse TNRC6 homologs of GW182 encode a PABP-interacting motif 2 (PAM2) in their C-terminal SD, which is important for interaction with PABPs (5,12,40). Furthermore, in zebrafish, miRNA-mediated silencing is mediated through two distinct motifs in the TNRC6 silencing motif, the canonical PAM2 and a P-GL motif. Whereas mutations in the PAM2 motif are sufficient for loss of interaction with PABP, only mutations in both motifs lead to a delay in miR-430-dependant deadenylation (40). *C. elegans* GW182 orthologs AIN-1 and AIN-2 lack PAM2 or PG-L motifs. In fact, AIN-1 and AIN-2 primary sequences diverge so much from GW182 that several authors still question whether they are homologs at all (43,44). Yet, our results presented here (see Figure 2) and early studies on AIN-1 and AIN-2 clearly establish their importance in miRNA-mediated silencing (14,18). Furthermore, a previous interaction study carried out in an heterologous system (15) showed that both AIN-1 and AIN-2 interact with ALG-1 as well as dmAGO1, and AIN-1 could interact with both *C. elegans* and *D. melanogaster* PABP, as well as deadenylase complexes PAN-2/3 and CCR4/NOT1. Hence, their functions and interactions indicate that even with a striking divergence at the level of primary sequence, Argonaute and PABP binding domains are encoded in *C. elegans* GW182 orthologs (15).

From an evolutionary standpoint, the exact mechanism through which miRNAs exert silencing may not be as important as the extent of gene silencing itself. Consequently, partial functional redundancy of miRNA-mediated translation silencing (whether poly(A)-dependent or -independent), deadenylation and decay, or their possible compensation for one another might have allowed tolerance of mutations that reorganize miRISC machinery and co-factors. As was highlighted in the present study, deadenylation-dependent and poly(A)-independent miRNA-mediated silencing mechanisms have now been observed across several organisms. This in turn suggests that significant selective pressure is being applied to retain multi-pronged aspects to the silencing mechanism, and that redundancy or compensation is partial at best. Therefore, discrete phenotypes may be uncovered specifically when impairing miRNA-mediated deadenylation, or poly(A)-independent silencing mechanisms. Exploiting the distinct requirements for deadenylation or translation repression may enable the disambiguation of the relative contributions of those two mechanisms in the biological functions of miRNAs. Their relative importance overall and the dependence on cellular and developmental context have been a matter of ongoing debate, but no genetic experimental design thus far allows to dissect the role of one mechanism from the other in living animals. This should now be achievable by exploiting genome-editing strategies that are becoming more accessible.

## Supplementary Material

SUPPLEMENTARY DATA
