# Supplementary material for: Poly(A)-binding proteins are required for microRNA-mediated silencing and to promote target deadenylation in C. elegans
Source: Nucleic Acids Res. 2016 Apr 19;44(12):5924–35. doi: 10.1093/nar/gkw276 (PMC4937315; doi:10.1093/nar/gkw276)
Supplement: SUPPLEMENTARY DATA [file supp_44_12_5924__index.html]

Poly(A)-binding proteins are required for microRNA-mediated silencing and to promote target deadenylation in C. elegans — SUPPLEMENTARY DATA 

# Poly(A)-binding proteins are required for microRNA-mediated silencing and to promote target deadenylation in *C. elegans*

## SUPPLEMENTARY DATA

- SUPPLEMENTARY DATA
