# Supplementary material for: Poly(A)-binding proteins are required for microRNA-mediated silencing and to promote target deadenylation in C. elegans
Source: Nucleic Acids Res. 2016 Apr 19;44(12):5924–35. doi: 10.1093/nar/gkw276 (PMC4937315; doi:10.1093/nar/gkw276)
Supplement: SUPPLEMENTARY DATA [file supp_gkw276_nar-02302-a-2015-File008.docx]

Inventory of supplemental information

File 1: Flamand et al. Supplementary text

Supplementary figures (5)

- Supplementary fig. 1
- Supplementary fig. 2 relates to Fig. 1
- Supplementary fig. 3 relates to Fig. 2
- Supplementary fig. 4 relates to Fig. 3
- Supplementary fig. 5 relates to Fig. 5

Supplementary Figure legends

Supplementary Material and methods

Supplementary Fig. 1,

Supplementary Fig. 2,

Supplementary Fig. 3,

Supplementary Fig. 4,

Supplementary Fig. 5,

**Supplementary Figure Legends**

Figure S1. PAB-1 and PAB-2 are conserved poly(A) binding proteins. (A) Phylogenic analysis of cytoplasmic poly(A) binding proteins in *C. elegans, Drosophila melanogaster, Danio rerio, Mus musculus, Homo sapiens*. (B) Percent identity matrix for various animal poly(A) binding proteins showed in (a) was generated using Clustal 2.1. (C) MLLE motif contains one substitution in both PAB-1 and PAB-2.

Figure S2. PAB-1/2 interaction with the miRISC is independent of RNA. (A) Characterization of the PAB-1/PAB-2 antiserum. Embryonic extracts from N2, *ok1851* (*pab-2* null) and *ok1851*; *pab-1* RNAi and N2;*pab-1* RNAi were probed with the PAB-1/2 antiserum. (B) A GST pull-down using either GST or GST-PAIP2 was performed either on WT (N2) embryonic extract in the presence of 0.1 ng/µL RNase A or 0.15µL MNase. The bound proteins were analyzed on SDS-PAGE and detected by western blot. (C) RNA from the RNase A and MNase treated extract was extracted and loaded on a 1.2% TAE-agarose and staining with ethidium bromide.

Figure S3. Screen for genetic interactors with the *let-7* miRNA. (A) *let-7(n2853)* animals (L1) were fed with bacterially-expressed dsRNA directed against the indicated gene and F1 animals were scored for bursting vulva phenotype at the permissive temperature (16°C). Results shown are representative of at least two experimental and biological replicates. (B) A diagram of p*lim-6* driven GFP expression in *C. elegans* larvae/adult. GFP is expressed in the ASEL neuron and excretory gland. Hypomorph mutants for *lsy-6* exhibit loss of GFP signal in ASEL neuron but not excretory gland in ~15% of animals. Pictures of an ASEL positive and negative animal were taken and DIC/GFP images were overlaid.

Figure S4. PAB-2 is dispensable for miRNA-mediated deadenylation. (A) *In vitro* translation extract was generated from *pab-2(0) ok1851* embryos and wild-type (N2) extract. A transcript containing RL, three binding sites for miR-35 and a poly(A)_86_ tail was incubated and deadenylation was monitored by UREA-PAGE.

Figure S5. Unadenylated reporter mRNAs do not undergo polyadenylation in the embryonic extract. (A) Design of the RNAs used in the experiment with the position of the oligonucleotides used for reverse transcription and qPCR are indicated. (B) Relative levels or RNAs in GST-treated and PAIP2-treated extract were detected by qRT-PCR using an oligo dT or internal RT primer after 0, 90 or 180 minutes of incubation. The presented results are representative of the 2 biological replicates.

**Supplementary Material and methods**

**GST-Pulldown with RNase A and MNase**

Embryos were homogenized in 3 volumes of lysis buffer (25mM Tris-Cl pH 7.5, 100mM KCl, 2.5mM MgCl_2_, 0.1% Triton X-100, 10% glycerol, 2mM CaCl2) using 30 strokes from a stainless steel homogenizer. 1.2 mg of S10 lysate was incubated with no RNase, 0.1ng/µL of RNase or 0.15U/µL microccocal nuclease for 20 minutes at 21°C. Reaction was stopped by adding 3mM of EGTA and pulldown was done with 50 µL of Gluthatione-Sepharose 4B beads (GE healthcare Life Sciences) pre-coupled to 75ug of GST-PAIP2 or GST recombinant protein for 1h30 at 4^o^C.­ Beads were washed 4 times in lysis buffer for 5 min at 4^o^C and bound proteins were eluted in 2x SDS loading buffer and analysed by SDS-PAGE and western blot.

***In vitro* Polyadenylation assay**

Standard *in vitro* deadenylation/stability assays were performed in presence of non-radiolabelled RNA, wherein 1 mM of RNA was used in 12.5µL reactions. Quiazol Isolated RNA was mixed with 500ng of N2 embryo total RNA and 100ng was used for reverse transcription with Superscript III using either oligo dT tdo679(GCGAGCTCCGCGGCCGCGTTTTTTTTTTTT) or internal primer tdo3160 (caacttgtttattgcagcttataatgg) for 1 hour at 50^o^C. cDNA was treated with RNaseH for 20 minutes at 37^o^C and diluted 1:100 in TE buffer. qPCR reaction was carried in technical triplicates (10µL) with BioRad SYBR Green Master mix and oligos tdo2384(TGGAGCCATTCAAGGAGAAG) and tdo2385(TGTAGTTGCGGACAATCTGG) using the Eppendorf MasterCycler RealPlex machine and software. Data was normalised to the value t=0min with the internal RT primer using the ΔΔCt method with an efficiency for the primer pair of 2.02.

**Phylogenic analysis.**

Protein sequences of flie, zebrafish, mouse and human PABPs were gathered from NCBI website and aligned using MacVector. Phylogenic tree was created by UPGMA method. Percent identity was calculated using Clustal 2.1.
